# Sexual conflict and the evolution of genitalia: male damselflies remove more sperm when mating with a heterospecific female

**DOI:** 10.1038/s41598-017-08390-3

**Published:** 2017-08-10

**Authors:** Adolfo Cordero-Rivera

**Affiliations:** ECOEVO Lab, Escola de Enxeñaría Forestal, Universidade de Vigo, Campus Universitario, 36005 Pontevedra, Galiza Spain

## Abstract

In *Calopteryx* damselflies, males remove rivals’ sperm stored by the female, thereby reducing sperm competition. This behaviour may create a sexual conflict, because females could lose the sperm stored in the spermatheca, used for long-term storage. Comparative evidence suggested antagonistic coevolution between sexes, which might prompt the evolution of narrow spermathecal ducts, or longer spermathecae, hindering sperm removal. *Calopteryx haemorrhoidalis* and *C. splendens* coexist and sometimes hybridize. Therefore, here I predicted that if females coevolve with conspecific males, heterospecific males should have an advantage when interspecific matings occur because females will show less resistance to them than to conspecific males. By hand-pairing females to males of both species, I found that in intraspecific and interspecific matings, sperm was almost completely removed from the bursa (97–100%), but only partially from the spermathecae, with more spermathecal removal in interspecific (63–71%) than intraspecific matings (14–33%). This suggests that heterospecific males are more efficient in sperm removal as predicted by a sexually-antagonistic coevolutionary scenario. Furthermore, in most cases, only the left spermatheca was emptied, suggesting that the evolution of more than one spermatheca might also be a female counter-adaptation to regain control over fertilization.

## Introduction

Animal genitalia was traditionally thought to be basically under natural selection^1^. Nevertheless, its evolution and diversification is clearly influenced by sexual selection acting on primary genital traits^2^. Eberhard^3^ was the first to clearly point out the weakness of lock-and-key mechanisms as the general explanation for the diversity of genitalia in animals. His suggestion of sexual selection as the main force behind genital evolution, elicited great interest in the topic reviewed by^4, 5^. The exact mechanism promoting genital diversification is nevertheless still under debate, but is unlikely to be the same in all species. In many cases, cryptic female choice^6^ seems the most pervasive force, although sperm competition^7^ and sexual conflict^8^ are also relevant mechanisms. In fact, these three ‘alternatives’ are sometimes very difficult to disentangle, and can be understood as a part of a continuum^5^. Recently, it has been suggested that sexual conflict and sexual selection are so intimately related that removing the first also removes the second, but eliminating sexual selection does not necessarily eliminates sexual conflict^9^. These ideas highlight the complexity behind the modern concepts of sexual selection, sexual conflict and cryptic female choice.

In the case of conflict for mating rate, the expectation is that males develop traits to increase mating frequency, and females counter-adaptations to reduce the costs of mating, such as the grasping antennae of some water striders and the anti-grasping apparatus of females^10^. The interaction between males and females during copulation is logically most intense between the male intromittent organ and the female genital opening. If there is conflict, male and female genitalia may coevolve in an arms-race scenario^11^, although evidence is still a matter of controversy^12^. By coevolution of male-female genital traits I refer here to changes in one sex apparently selected for to counteract specific genital characters of the other sex. I am not assuming a perfect match between the genitalia of both sexes, only that as one sex changes, the other also evolves as a response.

Three different methods have been used to test for coevolution between the sexes in their genitalia: comparisons between species in a lineage, manipulative experiments of genitalia and experimental evolution^13^. Only two studies have used cross-species matings to test for genital coevolution. In *Ohomopterus* carabid beetles, interspecific matings result in genital damages to males and females, due to mechanical incompatibilities, supporting a lock-and-key mechanism^14^. In *Drosophila*, micron-scale differences between species in their genitalia also result in wounding and infection to females, suggesting sexually antagonistic coevolution between the sexes^15^.

Here I conducted experimental copulations between species of the damselfly genus *Calopteryx* to study the mechanisms behind male-female coevolution of genitalia. In these damselflies, an elaborated pre-copulatory courtship prevents interspecific matings, which are rare even when two or more species coexist^16, 17^. *Calopteryx* species show little morphological differentiation in male genitalia, differing mainly in size^18^. The copulatory mechanisms of *Calopteryx* are well known. Females store sperm in a large bursa copulatrix and a “Y-shaped” spermatheca. At the moment of oviposition, the egg passing down contacts with a group of sensilla situated in the vaginal plates, and this elicits sperm ejection for fertilization^19^. Such communication between sensilla and the spermathecae has been corroborated when neural microsurgery of sensilla was carried out, which impeded spermathecal sperm emptying after sensilla stimulation^20^. Males have a penis head that removes sperm from the *bursa copulatrix* and two lateral horns, which are used to remove sperm from the spermatheca^21, 22^. During the first part of copulation (stage I), males remove sperm from the bursa and (in some species) also from the spermatheca, whereas in stage II the sperm is transferred to the female^23^. The spermatheca is used for long-term sperm storage, and in some species males apparently cannot remove sperm from this organ^19^, resulting in higher genetic diversity in spermathecal sperm^24^. For these reasons, the sperm in the spermatheca has been proposed as the centre of sexual conflict in *Calopteryx* ^25^. Males are expected to develop mechanisms to remove spermathecal sperm, and females are expected to evolve resistance, such as a reduction of spermathecal lumen to impede the introduction of male genitalia in that organ or an increase in spermathecal length^25^. Males could also elicit females to eject sperm by mimicking with their ligula the movements of eggs during fertilization, stimulating mechanical sensilla embedded in the female vaginal plates^26, 27^. This mechanism could be interpreted as evidence for cryptic female choice, due to male stimulation. If this were the case, then more sperm is expected to be ejected with increasing number of vaginal sensilla^28^.

In this paper, I bypass the precopulatory courtship by “hand-pairing”^29^ individuals of *C. haemorrhoidalis* and *C. splendens*. The studied population of *C. haemorrhoidalis* shows spermathecal sperm removal, with a minimum of sperm remaining after 60 movements of stage I^25^. The ability of males of *C. splendens* to remove spermathecal sperm is unclear, with evidence against^24^ and in favour^30^, and was therefore also addressed in this study. If sexually antagonistic coevolution is the main mechanism behind genital evolution, the expectation is that females should be able to resist conspecific male attempts to remove spermathecal sperm, and maintain control over fertilization. When females are mated to heterospecific males, the prediction is that males should be able to remove more sperm from the spermatheca, because in this situation, females could not evolve resistance mechanisms. If cryptic female choice is of relevance, then more sperm should be ejected by females having a higher number of vaginal sensilla.

## Results

### Anatomy of genitalia

**Figure 1:**
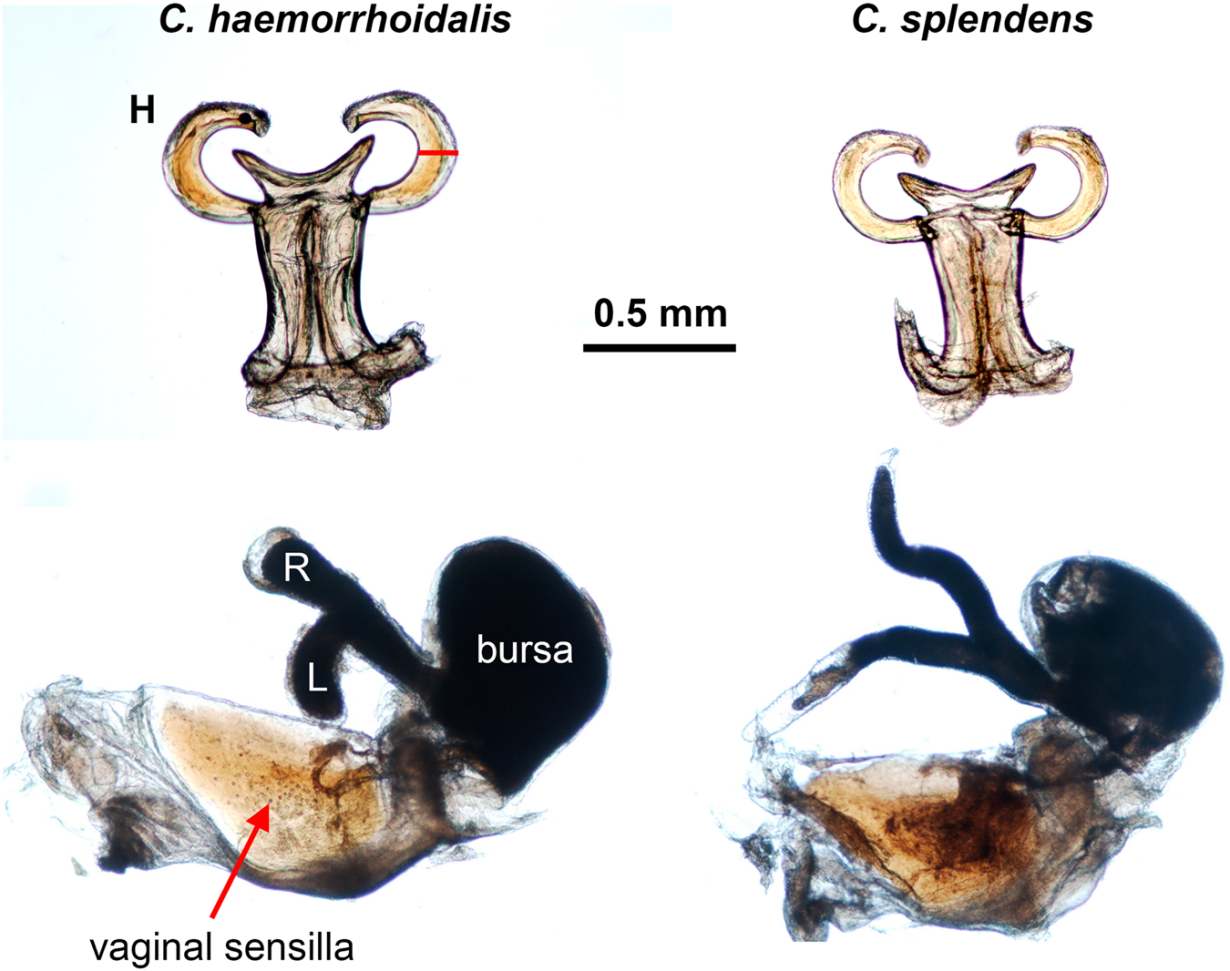
Male and female genitalia in *C. haemorrhoidalis* and *C. splendens*. The upper figures show the penis head in ventral view, with the lateral horns (H) used to remove sperm from the spermatheca. Note that the tri-dimensional position of the horns is not natural, because they were placed in a plane by a coverslip, to allow measuring their length. There were no significant differences between species in horn length (from the basis to the tip) and width (measured as indicated by the red segment on the middle of the horn). The lower figures show the female genital tract and sperm storage organs of mated females at the same scale as male genitalia. The sperm stored is the dark mass inside the bursa and the spermathecae. The spermathecae (L = left, R = right) were significantly longer in *C. splendens*, suggesting this is a counter-adaptation to difficult male sperm removal from that organ. (Scale bar = 0.5 mm, common for all images).

Male genital ligula ends in a head with two lateral processes (horns) covered by spines, and was very similar between the studied species (Fig. 1 and Table 1). In both species, the right horn was longer, but only significantly in *C. splendens* (Table 1).

**Table 1:** Biometry of male and female genitalia in *C. haemorrhoidalis* and *C. splendens* from Central Italy. Values are mean ± SE (N) (mm, except for number of sensilla).

Variable	*C. haemorrhoidalis*	*C. splendens*	Statistical test
Horn length (right)	0.620 ± 0.007 (18)	0.620 ± 0.007 (20)	Sides, *haemorrhoidalis*: F_1,15_ = 0.08, p = 0.775
Horn length (left)	0.617 ± 0.008 (18)	0.601 ± 0.009 (19)	Sides, *splendens*: F_1,18_ = 7.67, p = 0.013
Mean horn length	0.619 ± 0.006 (18)	0.611 ± 0.007 (20)	Species, F_1,35_ = 0.22, p = 0.642
Horn width (right)	0.098 ± 0.002 (18)	0.079 ± 0.002 (20)	Sides, *haemorrhoidalis*: F_1,15_ = 0.17, p = 0.682
Horn width (left)	0.097 ± 0.003 (18)	0.078 ± 0.001 (19)	Sides, *splendens*: F_1,18_ = 0.00, p = 0.985
Mean horn width	0.097 ± 0.002 (18)	0.079 ± 0.002 (20)	Species, F_1,33_ = 2.75, p = 0.107
Spermathecal length (right)	0.729 ± 0.028 (34)	0.953 ± 0.040 (38)	Sides, *haemorrhoidalis*: F_1,28_ = 2.20, p = 0.149
Spermathecal length (left)	0.714 ± 0.024 (33)	0.949 ± 0.034 (38)	Sides, *splendens*: F_1,34_ = 0.02, p = 0.894
Spermathecal length (mean)	0.724 ± 0.028 (32)	0.951 ± 0.030 (38)	Species, F_1,61_ = 50.23, p < 0.001
Spermathecal duct width	0.126 ± 0.005 (39)	0.117 ± 0.003 (48)	Species, F_1,70_ = 1.86, p = 0.177
Number vaginal sensilla	26.4 ± 0.6 (36)	31.3 ± 0.7 (46)	Species, F_1,74_ = 24.18, p < 0.001
Vaginal sensilla (right)	26.4 ± 0.6 (36)	31.0 ± 0.7 (46)	Sides, *haemorrhoidalis*: F_1,32_ = 0.02, p = 0.879
Vaginal sensilla (left)	26.3 ± 0.7 (36)	31.7 ± 0.7 (46)	Sides, *splendens*: F_1,43_ = 1.29, p = 0.263

Statistical tests are the results of ANOVAs comparing sides for the same species, with individual as a random factor, or comparing species. In all cases, wing length was entered as a covariate. Variables were Box-Cox or log transformed if needed, but the untransformed data are presented in the table.

Female genitalia was, nevertheless, clearly different between species, with *C. splendens* females having longer spermathecae (Fig. 1 and Table 1). In both species, the width of male horns is narrower than the width of the common duct of the spermatheca (Table 1), indicating that direct sperm removal from the spermathecae using male genital horns is possible in intra- and interspecific matings. Females of *C. splendens* had more sensilla in the vaginal plates than females of *C. haemorrhoidalis* (on average five more; Table 1, Fig. 1), and in both species there were no differences between sides. Therefore, if sperm is ejected by male stimulation, *C. splendens* females should expel more sperm.

### Copulatory behaviour

**Figure 2:**
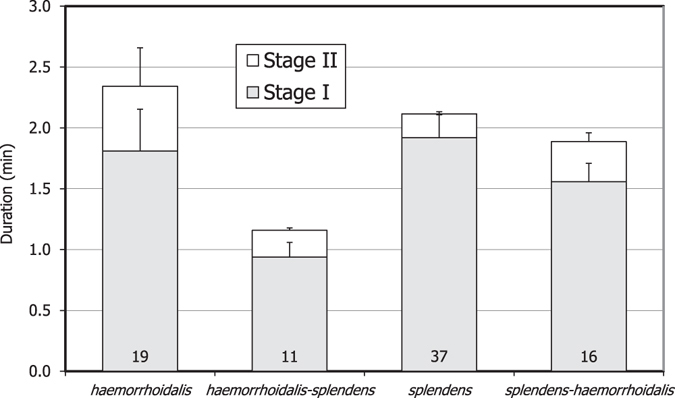
The mean duration of stage I (grey bars) and stage II (white bars; +SE) in intraspecific and interspecific matings between *C. haemorrhoidalis* and *C. splendens*. The sum of both stages indicates the total duration of copulation. In interspecific matings, the first name refers to the male and the second to the female. Numbers indicate sample size. Interspecific matings were significantly shorter, due to reduction in stage I, the phase when sperm removal occurs.

Copulation lasted 1.9–2.3 minutes in intraspecific matings, and also when males of *C. splendens* were mated to females of *C. haemorrhoidalis* (Fig. 2). Nevertheless, copulation was shortened to 1.2 minutes when males of *C. haemorrhoidalis* were mated to females of *C. splendens* (*h-s* matings). A two-way ANOVA was used to analyse the effect of male and female species and their interaction on copulation duration. In the case of stage I, there was a significant effect of the male species (F_1,79_ = 6.06, p = 0.016), with shortened duration by male *C. haemorrhoidalis* (Fig. 2). The female had no significant effect (F_1,79_ = 2.34, p = 0.130), but there was a highly significant effect of the male × female interaction (F_1,79_ = 9.52, p = 0.003), because interspecific matings were shorter. The duration of stage II was not significantly affected by male identity or the interaction (p > 0.209), although there was a marginally significant effect of female species (F_1,79_ = 3.17, p = 0.079).

The number of pumping movements during stage I was also affected by the male species (F_1,79_ = 12.05, p < 0.001), and the interaction (F_1,79_ = 8.19, p = 0.005), but not by the female (F_1,79_ = 0.74, p = 0.393). Intraspecific matings showed 75.3 ± 7.9 (19) movements in *C. haemorrhoidalis* and 100.5 ± 8.9 (37) in *C. splendens*. Interspecific *h-s* matings showed 47.0 ± 7.5 (11) movements, compared to 80.0 ± 8.9 (16) in *s-h* matings.

Half of *h-s* matings monitored (6 out of 11) had great difficulties to start, with several attempts before a secure genital contact was achieved. This mating direction was difficult to obtain, because many females did not cooperate, and refused to bring their abdomen in contact with male genitalia, and other pairs were unable to engage genitalia after several attempts and separated. On the other hand, in one mating between a male *C. splendens* and a female *C. haemorrhoidalis*, excluded from the above averages, copulation lasted 14.55 minutes, because the pair was for several minutes unable to disengage genitalia, indicating that also this mating direction may have some mechanical incompatibilities.

The volume of sperm in male’s vesicle was 0.068 ± 0.008 (14) mm^3^ in *C. haemorrhoidalis* and 0.053 ± 0.008 (17) in *C. splendens*. These values are 70–80% of the total volume of sperm of females after two intraspecific copulations: 0.084 ± 0.012 (6) in *C. haemorrhoidalis* and 0.074 ± 0.014 (8) in *C. splendens*.

### Sperm removal in *C. splendens*

**Figure 3:**
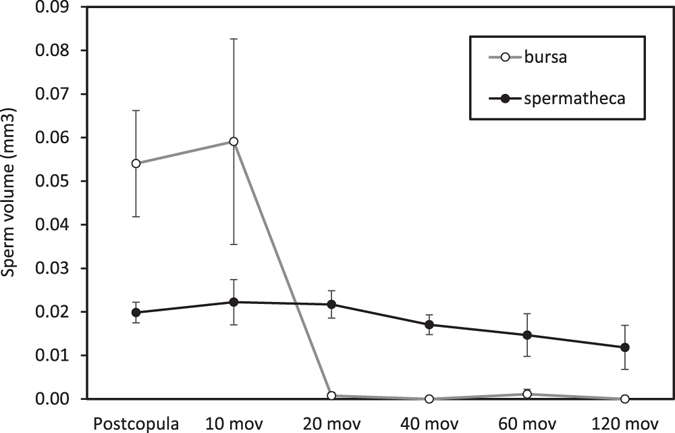
Sperm removal by male *C. splendens* in intraspecific matings. The volume of sperm stored in females preserved immediately after copulation (postcopula) is compared to the volume stored in double mated females, whose second mating was interrupted after a variable number of movements of the male genitalia during the stage I of copulation. Note that bursal sperm is completely removed after 20 movements. The volume of sperm in the spermatheca is negatively related to the number of stage I movements, but there are no significant differences among groups. Sample size is 8 for postcopula females and 5 for the other treatments.

The volume of sperm in the bursa copulatrix was drastically reduced (almost to zero) after only 20 movements of stage I in *C. splendens* matings (Fig. 3) and the differences were therefore highly significant among groups (ANOVA, F_5,27_ = 6.74, p < 0.001). The volume of the spermatheca was also reduced with increasing number of movements in the interrupted females (regression analysis, F_1,23_ = 4.34, p = 0.048), but the reduction was small, so that differences between groups were not statistically significant (ANOVA, F_5,27_ = 1.09, p = 0.388; Fig. 3).

### Sperm removal in intra- and interspecific matings

**Figure 4:**
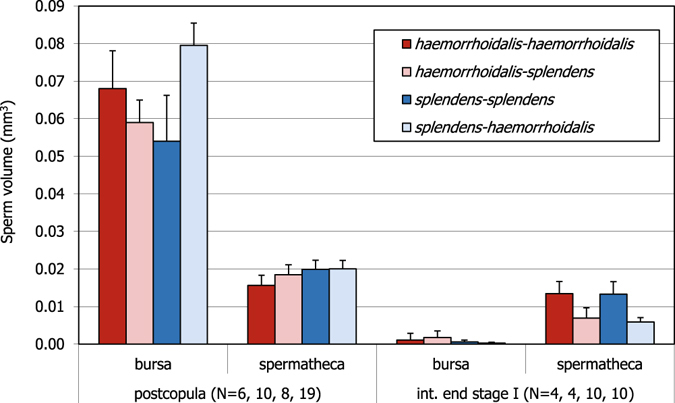
The volume of sperm in the *bursa copulatrix* and spermatheca of double mated females after intraspecific and interspecific matings (postcopula treatment; the male species is indicated first in the legend), compared to the volume stored by females mated to a conspecific male and then to a conspecific or heterospecific male, but interrupted at the end of stage I (60–120 movements), before insemination. The volume of sperm in the *bursa* is reduced by 97–100%. The volume of the spermatheca in intraspecific matings is reduced by 14% in *C. haemorrhoidalis* and 33% in *C. splendens*. In contrast, volume reduction is 63–71% in interspecific matings.

As expected, and corroborating previous experiments^25^, males of *C. haemorrhoidalis* were able to remove almost all (98%) the sperm stored in the bursa of conspecific females after 60 movements of stage I (Fig. 4). The same results were obtained for intraspecific *C. splendens* matings after 60–120 movements (99% of bursal sperm removal), and for both directions of interspecific matings (97–100% of bursal sperm removal, Fig. 4). Consequently, the effect of treatment (postcopula versus interrupted females) was highly significant (F_1,60_ = 128.72, p < 0.001). Intra- and interspecific matings removed a similar proportion of bursal sperm (F_1,60_ = 1.73, p = 0.194) and there was no treatment × type of mating interaction (intra- or interspecific; F_1,60_ = 1.17, p = 0.284).

In the case of the spermathecae, males removed part of the sperm stored (treatment effect: F_1,67_ = 22.49, p < 0.001). In intraspecific matings, males removed 14% in *C. haemorrhoidalis* and 33% in *C. splendens*, but in interspecific matings they were able to remove 63–71% (type of mating: F_1,67_ = 1.20, p = 0.276; treatment × type of mating interaction: F_1,67_ = 4.55, p = 0.037; Fig. 4). Furthermore, there was asymmetric sperm removal: the left spermatheca was emptied in 17 females, but only four females showed the right spermatheca empty (test for 1:1 proportion, z = 3.055, p = 0.002). Females were not asymmetric in sensilla number (Table 1). Therefore, this asymmetry in sperm volumes cannot be explained by sperm ejection.

**Figure 5:**
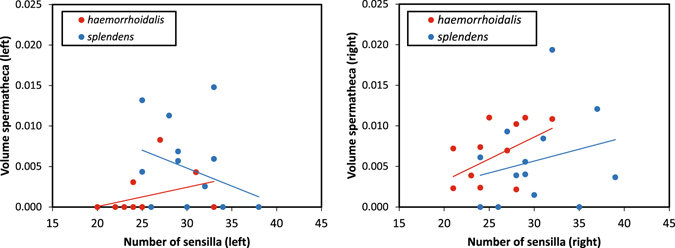
The relationship between the number of sensilla on the vaginal plates of female *C. haemorrhoidalis* and *C. splendens* and the volume of sperm (mm^3^) in the spermatheca of females whose second copulation was interrupted after 60–120 movements of stage I (N = 28 females). Note the different patterns between sides, the left spermatheca being emptied more frequently. On the left spermatheca, the effect of female species was significant. On the right spermatheca, the only significant (positive) effect was the number of sensilla. The species identity of the male had no significant effect in any case.

An ANOVA analysing the effect of species identity of the male and the female, their interaction and the number of sensilla as a covariate on the spermathecal volume after 60–120 movements (N = 28 females), suggests that both spermathecae are emptied with different efficiency (Fig. 5). On the left spermatheca, the only factor with a significant effect is female species (F_1,21_ = 4.53, p = 0.045), with a smaller sperm volume in *C. haemorrhoidalis* (Fig. 5). The species identity of the male, male × female interaction and the covariate had no significant effect (p > 0.140). On the right spermatheca, the volume of sperm remaining was positively correlated with the number of sensilla (F_1,20_ = 5.63, p = 0.028), with a marginally significant effect of male (F_1,20_ = 3.54, p = 0.075) and no effect of female species or the male × female interaction (p > 0.120; Fig. 5).

## Discussion

This experiment clearly shows that males of *Calopteryx* are able to remove sperm more efficiently when they mate with a heterospecific female, supporting a scenario of male-female coevolution in genital traits by sexual antagonism^2^. I have found no evidence of cryptic female choice by sperm ejection (see also ref. 30).

Experiments on artificial selection for high and low mating rates have shown that genitalia evolves very fast, supporting the antagonistic coevolution as one likely mechanism^31, 32^. The theory of sexually antagonistic coevolution is based on the premise that repeated interactions between sexes determine an arms-race due to the reciprocal evolutionary change that each sex imposes to the other^33^. Females may develop resistance to male adaptations, particularly when these produce injuries during sexual interactions, such as copulatory wounds^34^ or develop anti-clasping mechanisms^35^. Alternatively, females may develop tolerance mechanisms, to minimize the negative effects of male adaptations, such as behavioural^36^, anatomical^37^ and physiological responses^38^. My results indicate that in *Calopteryx* damselflies, genital coevolution is better explained by sexually antagonistic mechanisms, rather than by cryptic female choice. These results are in concordance with the higher success of heteropopulation (but conspecific) males in sperm competition experiments with the yellow dung fly (*Scatophaga stercoraria*), also interpreted as a case of sexually antagonistic coevolution due to allopatry^39^.

The genus *Calopteryx* has developed one of the most elaborate precopulatory courtships of damselflies, with males displaying wing colour, abdomen colour and oviposition substrates to potential mates^40^. Ethological divergence is clear^41^, but morphological difference negligible^18^. When *C. haemorrhoidalis* and *C. splendens* coexist in the same stream, a situation particularly common in Central Italy, interspecific interactions are frequent, but interspecific matings are rare, due to the ethological isolation promoted by precopulatory courtship. Nevertheless, some hybridization occurs^16^. My results indicate that heterospecific males may have higher fertilization success than conspecific males, if sperm volumes translate into fertilization rates, and may explain why some hybrid males are found in the field^16^.

It is interesting to note that males of the two *Calopteryx* species studied here show little divergence in the anatomy of the penis head, the part of the genitalia used to remove the sperm from previous matings^21^, a result which contrasts with the typically larger divergence in male genitalia between species^3^. Allopatric populations of *Calopteryx* diverge in their genital morphology more than in non-genitalic traits^25^, as expected from male-female coevolution. In some populations, males are able to physically remove sperm from the *bursa copulatrix* and the spermatheca, but in other populations, the spermathecal lumen is so narrow that direct sperm removal seems impossible^27^. In fact, a study of a French population of *C. xanthostoma* (considered sometimes as a subspecies of *C. splendens*) showed no evidence for spermathecal sperm removal^24^, while German populations of *C. splendens* do remove spermathecal sperm^30^, as the Italian population studied here. When males cannot physically remove spermathecal sperm, it has been shown that they sometimes exploit a pre-existent sensory bias in females by stimulating the vaginal sensilla (used by females in egg fertilization), which elicits sperm ejection by the female^42^.

In the two species studied here, male genital horns are narrower than the duct of the spermatheca of conspecific and heterospecific females, indicating that they can physically remove sperm from the spermathecae^25, 30^. Given that copulation duration is under male control in damselflies^43, 44^, one possible explanation for the short duration of interspecific copulations is that males emptied the spermatheca very fast, because only stage I is shortened (Fig. 2). The duration of stage II, when males inseminate, did not change. The sperm volume of the spermathecae was clearly reduced. This is easily explained by physical sperm removal. The possibility of sensory stimulation to elicit sperm ejection as the mechanism^42^, is not supported by the asymmetry in sperm removal, and the positive relationship between the volume of sperm remaining and the number of vaginal sensilla, when the expectation from this mechanism is a negative relationship^28^.

I have found clear differences in spermathecal sperm volume when comparing conspecific and interspecific matings (Fig. 4). In contrast with other interspecific copulations, where genital damage is common^34^, no evidence for genital damage was found in this experiment. Under the hypothesis of reciprocal evolution of genitalia by sexual selection, females are expected to coevolve with males and therefore be able to better resist in a scenario of sexual conflict^45^. In *Calopteryx*, the source of conflict is the sperm stored in the spermatheca^7^. In agreement with my predictions, males remove more sperm from heterospecific females, and this occurs in both directions. This pattern may be explained by antagonistic coevolution: females cannot coevolve with heterospecific males, and are therefore less able to resist to male manipulations. Males might also stimulate vaginal sensilla of heterospecific females more efficiently than conspecific males, and as a result females eject more sperm^26^, but I have found no evidence for this mechanism. In fact, even if the number of sensilla on *C. splendens* females is clearly higher than in *C. haemorrhoidalis*, there was no more sperm removal/ejection from this species (see below). Females of *C. splendens* have evolved long spermathecae, clearly longer than male genital horns, in contrast to *C. haemorrhoidalis*, where male and female genital sizes are almost identical. The evolution of these long spermathecae might be an example of a female counter-adaptation to make sperm removal more difficult^25^. The fact that males of *C. haemorrhoidalis* are able to remove 63% of spermathecal sperm of *C. splendens* females (after 60 movements) is therefore surprising, particularly compared to the 33% achieved by conspecific males (after 60–120 movements), and again suggests that sexually antagonistic coevolution is the explanation for this pattern.

Probably the most intriguing pattern is the asymmetrical sperm removal found in this experiment. This asymmetry had previously found in the same population of *C. haemorrhoidalis* ^25^ and more recently in *Calopteryx cornelia*, from Japan^46^. It seems that the morphology of penis head precludes the insertion of both horns simultaneously into the spermathecal common duct, and males specialise in using only one horn for spermathecal sperm removal^46^. The fact that left and right horns are similar in length (albeit the right one is longer) needs further research. The duplication of the spermatheca in *Calopteryx* might be another example of female counter-adaptation to male sperm removal ability^25^. It would be rewarding to examine whether asymmetric sperm removal is common in damselflies with Y-shaped spermatheca, which is a key prediction of this hypothesis.

## Methods

Fieldwork was carried out at two localities near Pontecorvo (Frosinone, Central Italy), where *C. haemorrhoidalis* and *C. splendens* have large populations, in August 2004. In the river Forma Quesa, *C. haemorrhoidalis* was the dominant species, whereas in the river Melfa, more specimens of *C. splenden*s were found. Both rivers are tributaries of the river Liri, and are separated by about 10 km. Damselflies were captured with a hand net, individually marked with and indelible pen, and introduced to an outdoor insectary (2 × 2 × 2 m), mounted on the shore of the river.

To obtain matings, females were hand-paired to a male^29^, by allowing the male to grasp the prothorax of the female with his anal appendages, and then releasing the female. Most pairs mated a few seconds after this manipulation, usually on the hand, which allowed a close observation of copulatory activity.

To study mating duration, females were hand-paired to a conspecific male (N = 19 females of *C. haemorrhoidalis* and 37 females of *C. splendens*) or to a heterospecific male (N = 16 females of *C. haemorrhoidalis* and 11 females of *C. splendens*) and allowed to complete copulation. For each mating, I measured the duration of the stage I, the number of pumping movements and the duration of stage II, to the nearest second. Using an ANOVA, the duration of stage I, stage II and number of movements were compared including the male species, the female and their interaction as explanatory variables. Response variables were normalised by a Box-Cox transformation before analysis.

To study sperm removal, a reciprocal hand-pairing experiment was designed. To control for previous female mating history, all females were first mated to a conspecific male. This was made to make sure that all females had sperm before the experimental mating. Previous research indicates that after 60 movements of stage I, males of *C. haemorrhoidalis* from this population have removed most of the sperm stored by females from previous matings^25^. In the case of *C. splendens*, the ability of males to remove bursal sperm was known for other populations^24^, but not for the study population. Therefore sperm removal was studied by comparing the volume of sperm stored by mated females (N = 8) with the volume remaining in females interrupted after 10, 20, 40, 60 and 120 movements of stage I (N = 5 in all cases) of their second mating.

After the first (intraspecific) mating, females were allowed to mate with a second male of the same species, or with a male of the other species, but copulation was interrupted after 60 movements of stage I in the case of male *C. haemorrhoidalis* (N = 4 females of each species). For *C. splendens*, 10 females interrupted after 60–120 movements from the previous experiment, were used for the intraspecific matings. A further group of 10 females of *C. haemorrhoidalis* were mated first to conspecific males and then to males of *C. splendens* and interrupted after 60–120 movements. In all cases, these copulations were interrupted before insemination. Specimens were immediately preserved in 70% ethanol and stored in individual plastic vials. To minimize the number of specimens used in the experiments, some males were used once as sperm donors and once as sperm removers. In the laboratory, females were dissected and the sperm storage organs extracted. In *Calopteryx*, these organs are a large *bursa copulatrix*, which receives the male genital ligula during copulation, and a “Y-shaped” spermatheca. Female genitalia were mounted on a slide and two insect pins of 0.2 mm of diameter placed over the slide, to allow the cover-slide to remain at this distance. The volume of sperm (in mm^3^) was estimated by calculating the area of the sperm mass using an image analysis software (ImageJ, https://imagej.nih.gov/ij/) and multiplying by 0.2 mm^47^. One sample was damaged during dissection and could not be measured. Final sample size for each group was 4–19 females (see Fig. 4). The volume of sperm was compared between treatments (mated females and experimentally interrupted females after 60–120 movements of stage I) and between intra- and interspecific matings using a two-way ANOVA with the interaction term. No transformation of sperm volume was needed to meet the normality assumption.

The sperm vesicle of 14 males of *C. haemorrhoidalis* and 17 males of *C. splendens* whose copulation was unsuccessful or interrupted during stage I was also dissected and the sperm mass mounted on a microscope slide, to measure its area and estimate volume. This represents the amount of sperm that males inseminate, because after copulation the sperm vesicle was empty (N = 4 males of *C. haemorrhoidalis* and 3 of *C. splendens*). Male genitalia was measured as in previous studies^48^. Results are reported as mean ± SE (N) throughout the text. Statistical analyses were done with xlStat (www.xlstat.com) and Genstat software^49^.
